# Lights-Transformer: An Efficient Transformer-Based Landslide Detection Model for High-Resolution Remote Sensing Images

**DOI:** 10.3390/s25123646

**Published:** 2025-06-11

**Authors:** Xu Wu, Xuqing Ren, Donghao Zhai, Xiangpeng Wang, Mehreen Tarif

**Affiliations:** 1 College of Computers Science and Cyber Security, Chengdu University of Technology, Chengdu 610059, China; 2 College of Geophysics, Chengdu University of Technology, Chengdu 610059, China

**Keywords:** remote sensing technology, landslides, deep learning, Lights-Transformer, GDCLD

## Abstract

In recent years, remote sensing technology has been extensively used in detecting and managing natural disasters, playing a vital role in the early identification of events like landslides. The integration of deep learning models has considerably enhanced the efficiency and accuracy of landslide detection particularly in automating the analysis and quickly identifying affected areas. However, existing models often face challenges, such as incomplete feature extraction, loss of contextual information, and high computational complexity. To overcome these challenges, we propose an innovative landslide detection model, Lights-Transformer, which is designed to improve both the accuracy and efficiency. This model employs an encoder–decoder architecture that incorporates multi-scale contextual information and an efficient attention mechanism, effectively capturing both local and global features of images while minimizing information loss. By introducing a Fusion Block for enhanced multi-angle feature fusion and a Light Segmentation Head to boost inference speed, Lights-Transformer extracts detailed feature maps from high-resolution remote sensing images, enabling the accurate identification of landslide regions and significantly improving detection accuracy. Compared to existing state-of-the-art landslide detection models, Lights-Transformer offers considerable advantages in accuracy, precision, and computational efficiency. On the GDCLD dataset, Lights-Transformer achieves an mIoU of 85.11%, accuracy of 97.44%, F1 score of 91.49%, kappa value of 82.98%, precision of 91.46%, and recall of 91.52%, demonstrating its exceptional performance.

## 1. Introduction

Landslides, as a type of sudden geological disaster, present a significant threat to human society, causing loss of life, damage to property, disruption of infrastructure, and ecological harm [1,2]. In recent years, the frequency of landslides has been continuously increasing, particularly in mountainous regions prone to earthquakes, such as China, Nepal, and Indonesia. Landslide disasters not only cause damage to the natural environment but also result in significant socio-economic losses. Therefore, how to accurately and efficiently detect and provide early warnings for landslides has become a crucial research topic in the field of disaster prevention and mitigation [3,4]. Traditional landslide detection methods primarily rely on geological surveys [5], on-site monitoring [6], and remote sensing image analysis [7]. Although these methods can provide reliable information on landslides to a certain extent, their generalizability and real-time capability are limited particularly in large-scale disaster monitoring and complex terrain conditions. They often involve high costs and long response times. Moreover, traditional methods usually rely on manual feature extraction, which makes it challenging to handle the high complexity and diversity in remote sensing images [8,9,10].

To overcome these challenges, deep learning-based methods for landslide detection in remote sensing images have garnered widespread attention in recent years [11,12,13,14,15]. The rise of convolutional neural networks (CNNs) [16,17] has made automatic feature extraction and semantic segmentation from remote sensing images feasible. Classic models such as U-Net [18], DeepLabv3 [19], HRNet [20], UperNet [21], and FCN [22] have been extensively applied in landslide detection tasks, yielding impressive results. Fang et al. [23] proposed a deep learning method based on LiDAR data for automatic landslide recognition, training a lightweight U-Net model. U-Net has achieved high F1 scores in landslide samples from the tropical mountainous regions of Colombia [24]. Huang et al. [25] improved the DeepLabV3 model by adding an attention mechanism module and modifying the spatial pyramid pooling module to enhance model accuracy. HRNet and UperNet, leveraging multi-scale architectures, have achieved over 90% pixel classification accuracy in complex terrain areas of southwest China [26,27]. Attention-UNet, by introducing an attention mechanism, has achieved F1 scores of up to 80% in certain earthquake-affected areas [28]. Chen Xuerong et al. [29] adopted a fully convolutional network (FCN-FL) with focal loss to draw historical landslides in imbalanced regions, improving feature extraction and reducing background loss. These models effectively extract terrain, texture, and other features from remote sensing images using multi-layer convolution structures, enabling the precise segmentation of landslide areas. However, CNN models still face challenges, such as limited ability to capture long-range dependencies and inadequate performance in recovering boundary details, particularly for irregular and small landslide targets. Additionally, with increasing network depth, CNN models experience significant computational complexity and memory consumption, which hinders real-time processing, especially for high-resolution remote sensing images.

However, existing methods still encounter several critical challenges in practical applications. First, CNN architectures are limited by local receptive fields, which makes it difficult to capture long-range feature dependencies. This limitation results in reduced detection accuracy, particularly for small-scale or boundary-complex landslide targets. Second, the generalization capability of these models under varying terrain and illumination conditions is limited, making them prone to overfitting. Third, when processing high-resolution images, the computational complexity increases significantly, which restricts the deployment of these models in real-time or large-scale application scenarios.

To address these limitations, Transformer models [30,31] have been introduced into the field of remote sensing image processing. For example, Zhao et al. employed a Transformer-based approach for landslide susceptibility mapping, significantly improving boundary detection and feature extraction performance [32]. The SCANet model combines a Transformer encoder with a CNN decoder, resulting in an mIoU improvement of over 1.95% on a customized landslide dataset [33]. In addition, two recent studies have demonstrated the effectiveness of different models for landslide detection tasks. Yang et al. proposed a detection model based on ResU-Net, which incorporates a Transformer into the ResU-Net structure to enhance the network’s capability of modeling global contextual information in feature maps and drive the model to identify landslides using small datasets [34]. Another study employed the SegFormer model, which utilizes a Transformer encoder to extract multi-scale features from high-resolution remote sensing images. It achieved an mIoU exceeding 75% in complex terrain, fully demonstrating the potential of the Transformer architecture in landslide detection [35].

Although numerous landslide detection methods based on remote sensing image processing have achieved some success, several key challenges remain unresolved. First, in scenarios characterized by complex terrain and severe illumination variations, existing methods have limited local feature extraction capabilities, which leads to poor generalization and an inadequate stability of detection results. Second, processing large-scale remote sensing images comes with high computational costs and memory demands, limiting their applicability in real-time scenarios. Furthermore, when dealing with irregular boundaries of landslide areas, current models are prone to misdetections or boundary blurring, which reduces the reliability of detection results. Additionally, most existing methods rely on two-dimensional image data and fail to effectively model three-dimensional terrain structures, which impacts detection accuracy under specific geomorphological conditions. These unresolved challenges form the primary motivation behind the Lights-Transformer model proposed in this study.

To address these challenges, this study proposes a novel Transformer-based model for landslide detection called Lights-Transformer. The model integrates an advanced self-attention mechanism and an efficient encoder–decoder architecture to better capture long-range dependencies and provide global information modeling, making it particularly suitable for complex terrain landslide detection tasks. Although this architecture has been widely applied in various image segmentation tasks, it still exhibits significant advantages in landslide detection tasks. On one hand, landslide regions in remote sensing images often exhibit characteristics such as small targets, blurred boundaries, and complex terrain. The encoder progressively extracts high-level semantic information, enhancing the discrimination capability for complex landslide areas. On the other hand, the decoder restores spatial resolution layer by layer and combines multi-scale features for semantic reconstruction, which facilitates the fine delineation of landslide boundaries. Specifically, Lights-Transformer introduces two key modules: the Fusion Block, which enhances feature extraction through multi-scale context integration, and the Light Segmentation Head, which reduces computational complexity while maintaining high detection precision. Therefore, the proposed model does not simply reuse a conventional structure but instead carefully considers the specificity of landslide detection tasks and practical application requirements in its structural design and module composition.

The main contributions of this study are as follows:

1. A novel Transformer-based landslide detection model, Lights-Transformer, is proposed to address the limitations of traditional methods in handling complex terrains and high-resolution images;
2. The introduction of Fusion Block and Light Segmentation Head modules enhances the model’s detection accuracy and inference efficiency through feature fusion and lightweight design;
3. A comparison with state-of-the-art models (such as U-Net, DeepLabv3, HRNet, etc.) demonstrates the advantages of Lights-Transformer in landslide detection tasks.

The remainder of this paper is organized as follows: Section 1 presents the study area and data; Section 2 provides a detailed description of the Lights-Transformer model architecture; Section 3 demonstrates experimental design and result analysis; Section 4 presents the Analysis and Discussion; Section 5 concludes this study and proposes future research directions; finally, Section 6 presents the conclusions of this study.

## 2. Study Area and Data

In this study, we selected the publicly available GDCLD landslide dataset [36] for training and testing the proposed model. The GDCLD landslide dataset is a comprehensive dataset that integrates multi-source remote sensing imagery, including PlanetScope, Gaofen-6, Map World, and Unmanned Aerial Vehicle (UAV) data.

It covers landslides triggered by nine global earthquakes from various geographical and geological backgrounds. The GDCLD dataset is freely available at https://doi.org/10.5281/zenodo.13612636 accessed on 24 October 2024.

**Figure 1:**
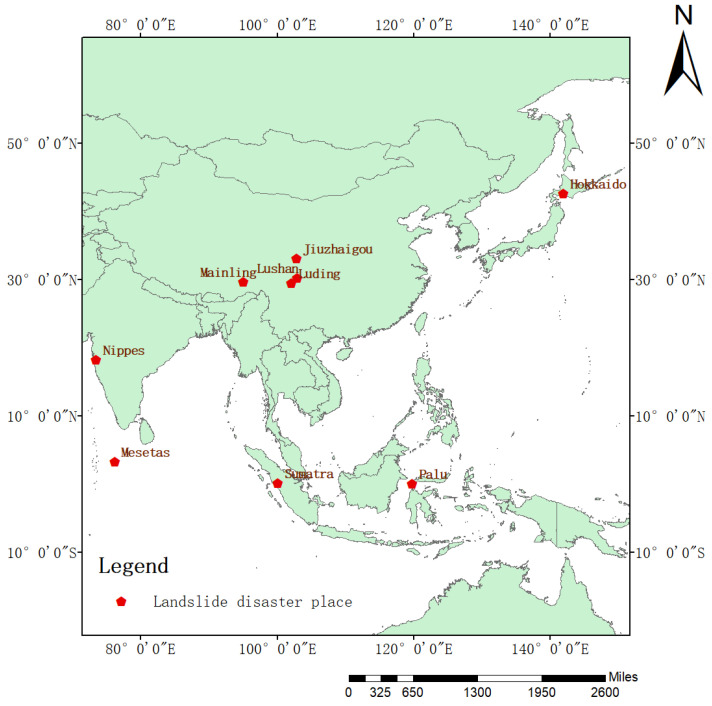
Study area.

This dataset includes landslides caused by earthquakes in nine regions: Jiuzhaigou, Mainling, Hokkaido, Palu, Mesetas, Nippes, Sumatra, Lushan, and Luding. Table 1 presents detailed information on this landslide dataset, and Figure 1 shows the specific locations of these regions.

Although the GDCLD dataset integrates multi-source remote sensing images from nine global earthquake-induced landslide regions, providing high scalability and diversity, it still presents certain potential biases in regional distribution. Landslide occurrences are influenced by a combination of geomorphological characteristics, climatic conditions, and human activities. If model training and evaluation are conducted solely based on specific geomorphologies (e.g., mid-to-high mountainous areas), it may lead to overfitting and reduce generalization performance in unseen regions (such as low hills, extreme arid areas, or permafrost zones). In particular, the GDCLD dataset includes regions with certain similarities in vegetation cover and rainfall patterns, which may interfere with the model’s ability to adapt to complex and variable terrain conditions.

During the data preprocessing stage, all remote sensing images were uniformly cropped into 1024 × 1024 pixel patches using a non-overlapping cropping strategy to accommodate the input requirements of deep models and to facilitate tiling and reconstruction during the testing phase, thereby enhancing the model’s robustness and generalization ability across different spatial scales.

In addition, to comprehensively evaluate the model’s adaptability across different terrains, this study selected four representative test regions from the GDCLD dataset: Lushan (plateau hills), Palu (tropical mountains), Mesetas (arid plateau), and Sumatra (tropical rainforest). These regions exhibit significant differences in geomorphological structure, landslide density, and disaster morphology, covering typical natural geographical conditions, which is conducive to testing the stability and practicality of the Lights-Transformer model in various geographical environments.

**Figure 2:**
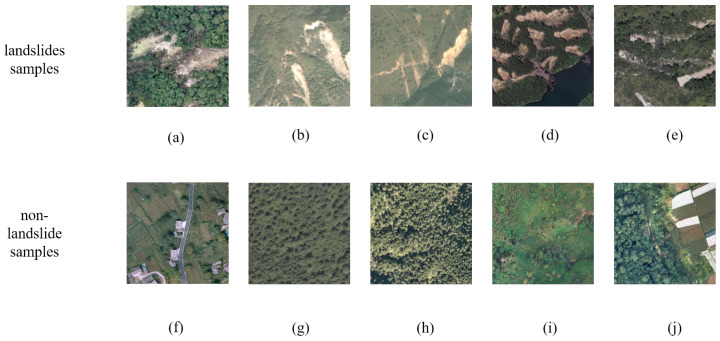
(**a**–**e**) Landslide samples; (**f**–**j**) non-landslide samples.

Figure 2 illustrates some typical landslide and non-landslide samples from the dataset.

## 3. Methods

**Figure 3:**
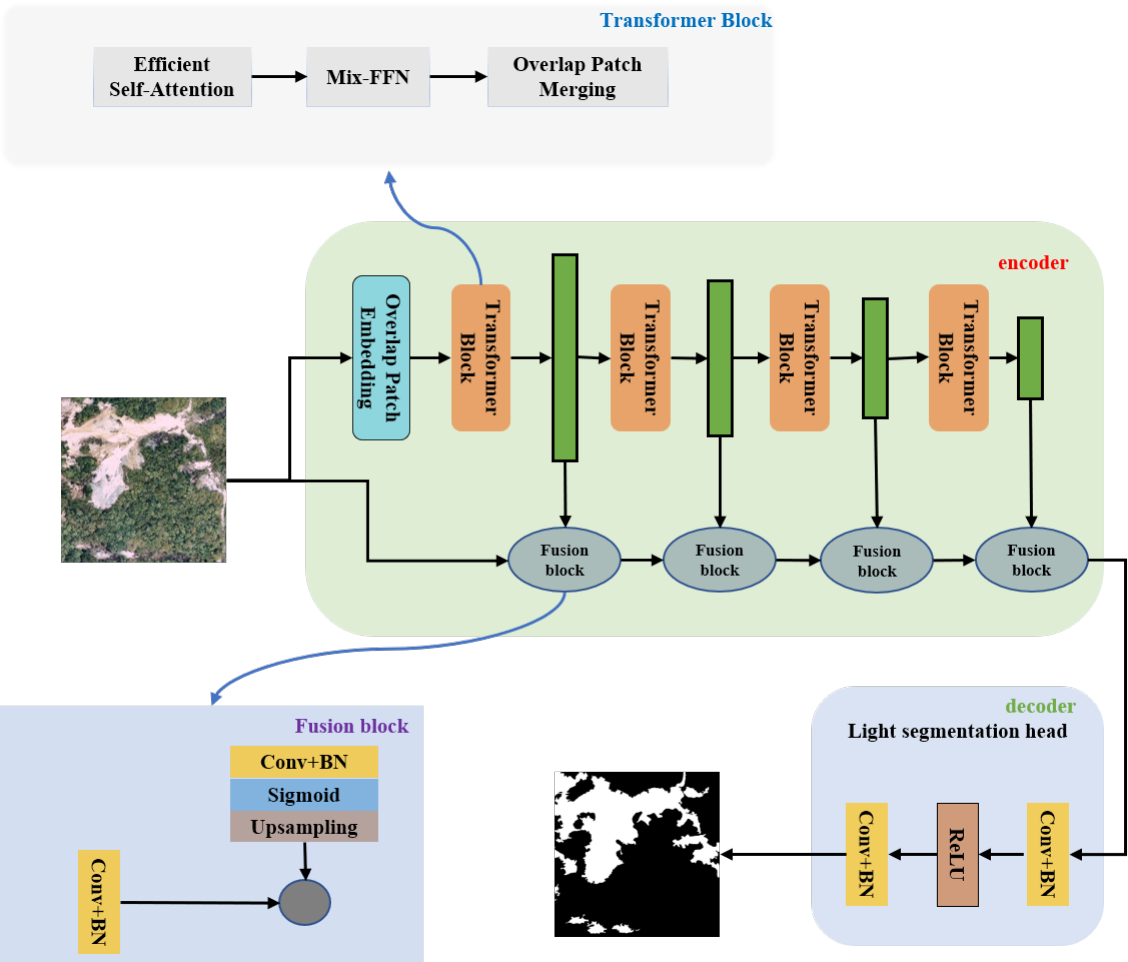
Lights-Transformer model architecture.

This study proposes a landslide detection model based on the Transformer architecture named Lights-Transformer. The model combines the self-attention mechanism with an efficient encoder–decoder structure, enabling feature learning through multi-layer Transformer encoders, thereby achieving exceptional scalability and adaptability. The core of the model includes modules such as Overlap Patch Embedding, Efficient Self-Attention, Mix-FNN, Overlap Patch Merging, Fusion Block, and the lightweight Segmentation Head. These modules work synergistically to enhance feature extraction capabilities and computational efficiency. The following Figure 3 illustrates the architecture of the proposed method.

The main components of the Transformer include the Overlap Patch Embedding, Efficient Self-Attention, Mix-FNN, and Overlap Patch Merging layers.

### 3.1. Overlap Patch Embedding

Since landslide regions are often irregularly distributed and contain rich terrain textures and local features, the use of an “overlapping cropping” strategy during the image preprocessing stage allows for the preservation of more edge information and enhances the perception capability of small-scale landslide areas. In Lights-Transformer, the image is first divided into multiple overlapping patches with each patch generating an embedding vector through a convolution operation. The stride setting of the convolution controls the overlap ratio of the patches, and the overlapping regions between adjacent patches provide the model with additional contextual information. This design helps capture local features of the image and enhances the continuity of spatial features. Particularly when dealing with high-resolution images, it aids in retaining detailed information and mitigating information loss. We used uniformly cropped 1024 × 1024 image patches as inputs during both model training and inference phases to ensure consistency in image dimensions and to avoid gradient instability issues caused by cross-scale training.

### 3.2. Efficient Self-Attention

To effectively extract features from image patches, we employ a lightweight self-attention mechanism: namely, the Efficient Self-Attention (ESA) layer. The design intention of ESA is to address the high computational complexity and memory overhead associated with the traditional multi-head self-attention mechanism of Transformers when processing high-resolution remote sensing images. Unlike traditional multi-head self-attention, ESA significantly reduces computational load and the number of parameters, thereby improving the model’s efficiency when processing high-resolution images and effectively alleviating overfitting issues. In traditional self-attention mechanisms, calculating the similarity between each pair of patches requires a full computation, with a complexity of $O(N^{2})$, where *N* is the length of the image sequence. To reduce computational complexity, ESA introduces an approximation ratio *R* to shorten the length of the input sequence, thereby reducing the computational complexity from $O(N^{2})$ to O(NR).

The formula for the traditional self-attention mechanism is shown in Equation (1).

(1) $$Attention\left(Q,K,V\right)=Soft\max\left(\frac{QK^{T}}{\sqrt{d_{head}}}\right)V,$$

where *R* is the approximation ratio used to adjust the computational complexity, and *K* is reshaped and linearly transformed using the following Equation (2):

(2) $$K^{\prime }=Linear\left(K\right),$$

Through this method, ESA reduces the computational complexity in terms of dimensions and significantly improves processing efficiency in practical applications.

### 3.3. Mix-FNN

To effectively handle image positional information, we introduce Mix-FNN, which avoids the limitations of traditional position encoding (PE) with fixed resolutions. The Mix-FNN module aims to address the sensitivity of the Transformer’s fixed positional encoding to the input image resolution. In ViT, position encoding is typically fixed, and when the resolution during the test phase differs from that in the training phase, interpolation is required, often leading to a decrease in accuracy. Mix-FNN implicitly learns patch positional features by introducing a 3 × 3 convolutional layer in the feedforward network and directly adds the positional information to the feature map. Specifically, Mix-FNN combines a 3 × 3 convolution with a multilayer perceptron (MLP). When the input feature is $X\in R^{N\times d}$, the output *Y* of Mix-FNN is given by the following Equation (3):

(3) $$Y=MLP(Conv_{3\times 3}\left(X\right)),$$

where $Conv_{3\times 3}$ represents the 3 × 3 convolution operation, and MLP is the multi-layer perceptron used to further process the positional embedding information obtained through the convolution.

### 3.4. Overlap Patch Merging

In remote sensing image landslide detection tasks, landslide regions often span multiple patches, exhibit fragmented boundary contours, and are highly prone to boundary discontinuity during patch partitioning, which leads to model misjudgments. To address this, we introduced the Overlap Patch Merging module. This module analyzes feature similarity and class consistency between adjacent patches to perform spatial rearrangement and boundary smoothing at the feature level, effectively alleviating semantic fragmentation caused by patch-based processing. Compared with conventional simple stitching or channel concatenation strategies, Overlap Patch Merging is more suitable for continuous region modeling in high-resolution remote sensing images, improving the discriminative performance of landslide edges and background transition areas. In remote sensing images, adjacent regions often exhibit semantic continuity, such as landslides typically expanding along mountain slopes. This module enhances spatial consistency by intelligently merging adjacent patches, reducing the issue of “broken” landslide boundaries.

### 3.5. Fusion Block

Landslide regions in remote sensing images often exhibit complex boundaries and significant scale variations with strong spatial extensibility and both local texture and global semantic information being important. Single-scale feature extraction methods struggle to comprehensively capture the structural characteristics of landslides, which can lead to blurred boundaries or missed detections. To address this, we designed the Fusion Block module, which integrates feature maps from different resolutions. The purpose of the Fusion Block is to enhance the multi-scale fusion capability between high- and low-resolution features. Unlike traditional feature concatenation or direct additive fusion, the Fusion Block introduces a semantic-guided attention mechanism. This mechanism extracts semantic weights from low-resolution features to weight and enhance high-resolution features. This approach improves boundary clarity while significantly enhancing the model’s ability to detect small, irregularly shaped landslide regions, addressing the issue of severe information loss in complex terrains associated with traditional fusion methods. When processing high-resolution feature maps, we introduce the Fusion Block module. This module extracts high-resolution features through a 1 × 1 convolution and batch normalization (Batch Normalization) layers. Low-resolution feature maps are then passed through 1 × 1 convolution, batch normalization, and a Sigmoid layer for processing. Afterward, bilinear interpolation is used for upsampling to generate semantic weights. The semantic weights are element-wise multiplied with the high-resolution feature maps, thereby combining the semantic information of the low-resolution image with the features of the high-resolution image.

Specifically, the output of the Fusion Block is obtained through the following Equations (4) and (5):

(4) $$S=\sigma (Conv_{1x1}\left(F_{low}\right)),$$

(5) $$F_{fusion}=S\cdot F_{high},$$

where $F_{low}$ and $F_{high}$ represent the low-resolution and high-resolution feature maps, *S* is the semantic weight obtained through the Sigmoid function, and $F_{fusion}$ is the fused feature map.

### 3.6. Light Segmentation Head

In remote sensing scenarios, landslide regions typically occupy a small proportion of the image and exhibit diverse morphologies. Therefore, the segmentation head needs to possess high-resolution classification capability while maintaining inference efficiency. To this end, following the final Fusion Block, we designed a lightweight segmentation head that uses two convolutional layers combined with BatchNorm. To improve inference speed, the lightweight segmentation head optimizes computational efficiency while maintaining high accuracy, reducing the number of parameters and enabling the rapid generation of refined masks to achieve a balance between accuracy and speed. Despite its simplicity, we ensured sufficient representation capacity by adjusting the convolution kernel sizes and channel numbers, enabling this module to effectively distinguish between landslide and background regions. In practical experiments, this module maintained high accuracy while keeping the inference time under 103 ms, demonstrating excellent task adaptability and computational cost-effectiveness.

Overall, the modules of the Lights-Transformer model collaborate and complement each other, significantly enhancing the accuracy and efficiency of landslide detection: Overlap Patch Embedding preserves the spatial continuity and detailed features of images; ESA provides efficient global context modeling capabilities; Mix-FNN enhances the model’s local spatial positional awareness; Overlap Patch Merging achieves semantic alignment across multi-scale features; Fusion Block finely integrates semantic information from high- and low-resolution features; and the Light Segmentation Head greatly improves inference speed while maintaining accuracy. These six modules collectively constitute a compact and high-performance landslide detection framework, enabling Lights-Transformer to achieve precise landslide area recognition in complex terrains and high-resolution remote sensing images, demonstrating excellent robustness and practicality, especially in scenarios characterized by complex boundaries and densely distributed small targets.

## 4. Experiments

### 4.1. Experimental Design

**Figure 4:**
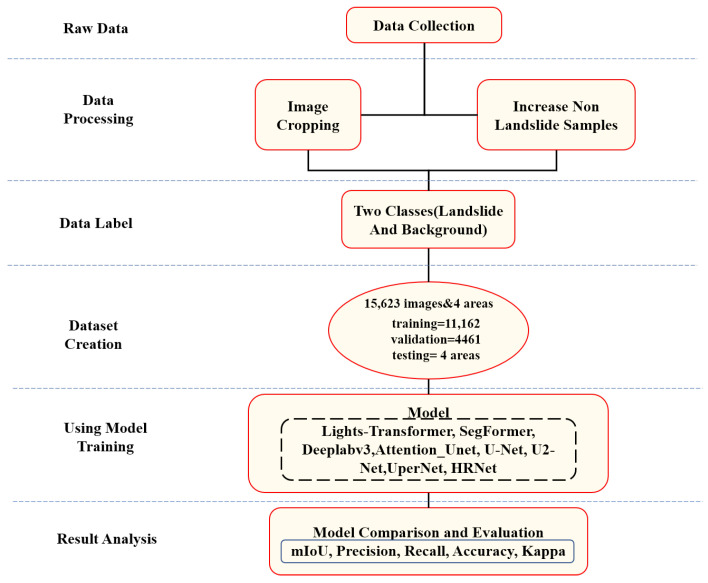
Flowchart of the experiment.

This study uses images obtained through remote sensing technology to detect landslide areas. The proposed model, Lights-Transformer, is employed for landslide detection, and it is trained and tested on the GDCLD landslide dataset. The performance of our model is compared with existing deep learning-based landslide detection models, including Attention-UNet [37], Deeplabv3, HRNet, SegFormer [38], U-Net, U2-Net [39], and UperNet. The workflow of this study is executed following the steps outlined below, and Figure 4 illustrates this process:

1. Data Preprocessing: The data are split into a training set and a validation set in a 3:1 ratio, while the data from the remaining four regions are used as the test set to evaluate the model’s generalization ability. Both the training and validation sets consist of images of size 1024 × 1024 pixels, and the test set corresponds to the entire region size. A custom algorithm was designed to crop the remote sensing images into 1024 × 1024 pixel-sized, non-overlapping patches for model testing;
2. Model Training: The model is trained using remote sensing images and their corresponding masks from the training set. The number of iterations for all models is set to 30,000;
3. Model Validation: During the training process, the model is validated every 500 iterations. The model’s parameters are optimized to reach the best configuration;
4. Model Prediction: The model is tested on various regions from the test set, and the individual image patches are stitched together to generate the landslide predictions for the region.

In summary, we present the training process of the model in Algorithm 1 as follows. The training data and validation data are inputted, with a batch size of 2 for each iteration, and the model is trained for 30,000 iterations. Finally, the test set is used for prediction with the trained model.
**Algorithm 1:** Landslide detection of Lights-Transformer
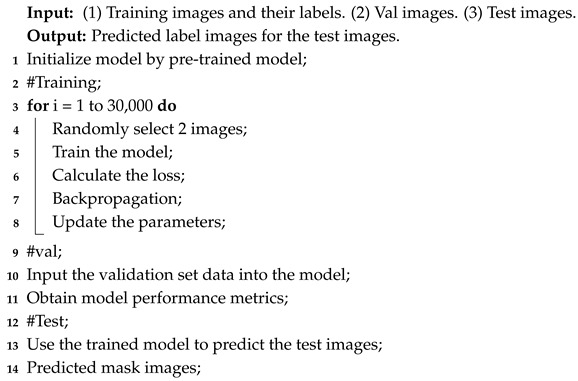


### 4.2. Experimental Setup

We present the hardware platform and environment parameters used in the experimental process in Table 2 below.

The key parameters used during the experiment are listed in Table 3.

In this study, model training and testing were conducted on a workstation equipped with an NVIDIA GeForce RTX 3090 GPU (24 GB VRAM) and an Intel Xeon Gold 6226R CPU, running Windows 11, using the PaddlePaddle deep learning framework. Based on the GDCLD dataset, the batch size was set to 2, and approximately 8 h were required to complete 30,000 iterations. During the inference phase, the Lights-Transformer model achieved an average processing time of only 103 milliseconds per 1024 × 1024 pixel image, demonstrating high computational efficiency and indicating that the model is capable of efficient and scalable real-time landslide detection deployment on mainstream GPU platforms.

### 4.3. Evaluation Metrics

To conduct a systematic comparison, we employed five widely used metrics to evaluate the performance of all semantic segmentation models: mIoU, precision, recall, F1 score, accuracy, and kappa. These metrics provide a comprehensive assessment of the models’ capabilities in accurately segmenting landslide regions.

mIoU is a metric used to evaluate the performance of image segmentation models, measuring the degree of overlap between the predicted segmentation and the ground truth labels.

(6) $$\mathrm{IoU}=\frac{\mathrm{TP}}{\mathrm{FN}+\mathrm{FP}+\mathrm{TP}}$$

(7) $$\mathrm{mIoU}=\frac{1}{2}\left({\mathrm{IoU}}_{\mathrm{landslides}}+{\mathrm{IoU}}_{\mathrm{backgrounds}}\right)$$

Precision is used to determine the correctness of landslide area detection.

(8) $$\mathrm{Precision}=\frac{\mathrm{TP}}{\mathrm{TP}+\mathrm{FP}}$$

Recall is used to define how many actual landslide areas are identified in the image.

(9) $$\mathrm{Recall}=\frac{\mathrm{TP}}{\mathrm{TP}+\mathrm{FN}}$$

Accuracy is the percentage of correctly predicted data out of all data.

(10) $$\mathrm{Accuracy}=\frac{\mathrm{TP}+\mathrm{TN}}{\mathrm{TP}+\mathrm{TN}+\mathrm{FP}+\mathrm{FN}}$$

The balance between precision and recall is calculated using the F1 score.

(11) $$F1=\frac{2\times \mathrm{Precision}\times \mathrm{Recall}}{\mathrm{Precision}+\mathrm{Recall}}$$

The kappa coefficient is used to measure the consistency between landslide and non-landslide areas.

(12) $$\begin{array}{c} P_{e}=\left(\frac{\mathrm{TP}+\mathrm{FP}}{N}\times \frac{\mathrm{TP}+\mathrm{FN}}{N}\right)+\left(\frac{\mathrm{TN}+\mathrm{FN}}{N}\times \frac{\mathrm{TN}+\mathrm{FP}}{N}\right) \end{array}$$

(13) $$\begin{array}{c} \mathrm{Kappa}=\frac{\mathrm{Accuracy}-P_{e}}{1-P_{e}} \end{array}$$

where TP (True Positive), FP (False Positive), TN (True Negative), and FN (False Negative) can be calculated using the confusion matrix (see Table 4), and N represents the number of samples. The overall performance is evaluated using the average IoU, precision, recall, accuracy, and F1 score for both the landslide and background classes. For all metrics in the above formulas, the higher, the better.

### 4.4. Comparison of Landslide Detection Accuracy

In this study, the effectiveness of the Lights-Transformer model was compared with models from other application domains. During the experiments, our model was trained and tested alongside other well-known models under the same conditions to objectively assess the performance of Lights-Transformer. The same dataset was used for processing. Table 5 shows the performance evaluation metrics of these models tested on the research set.

As shown in the Table 5, the proposed Lights-Transformer model demonstrates significant advantages in landslide detection tasks. The experimental results indicate that Lights-Transformer outperforms across key performance metrics, including mean Intersection over Union (mIoU, 85.11%), accuracy (97.44%), F1 score (91.49%), kappa coefficient (82.98%), precision (91.46%), and recall (91.52%). These results clearly demonstrate the exceptional overall segmentation accuracy and category differentiation capabilities of Lights-Transformer.

As a core metric for evaluating segmentation models, mIoU reflects the model’s ability to distinguish between different categories. Lights-Transformer achieves an mIoU of 85.11%, significantly surpassing the second-best model, SegFormer (83.13%), with an improvement of 1.98 percentage points. This advantage highlights the superior boundary refinement and single-pixel classification accuracy of Lights-Transformer. Furthermore, compared to other mainstream models such as HRNet, UperNet, and Deeplabv3, Lights-Transformer exhibits a more pronounced improvement in mIoU, underscoring the Transformer model’s ability to capture global contextual information and handle complex features. In contrast, traditional convolutional networks such as U-Net and Attention-UNet show significantly inferior performance, while ResU-Net achieved an mIoU of 77.50%, which is at an upper–medium level.

Accuracy measures the correctness of the model in overall pixel classification. Lights-Transformer achieves the highest accuracy of 97.44%, demonstrating its ability to accurately classify landslide regions and background areas, ensuring high reliability. In comparison, while SegFormer achieves an accuracy of 97.09%, Lights-Transformer improves by 0.35 percentage points, showcasing stronger global modeling capabilities and classification robustness. Moreover, compared to traditional models such as U-Net (92.95%) and Deeplabv3 (95.76%), Lights-Transformer’s advantage in accuracy is even more evident, further highlighting its superior performance.

The F1 score, which combines precision and recall, is an important metric for evaluating segmentation models. In this study, Lights-Transformer achieves an F1 score of 91.49%, significantly higher than SegFormer (90.17%) with an improvement of 1.32 percentage points, demonstrating its precise capture of landslide regions while effectively reducing false positives. Additionally, Lights-Transformer improves by approximately 2 percentage points compared to HRNet (88.93%) and UperNet (89.26%). Traditional models such as U-Net (76.02%) and Attention-UNet (78.81%) show significantly lower F1 scores, particularly struggling with complex landslide boundary recognition.

The kappa coefficient measures the agreement between classification results and ground truth, accounting for random classification effects. Lights-Transformer achieves the best kappa coefficient of 82.98%, indicating higher consistency in classifying landslide and background regions. By comparison, the second-best model, SegFormer, achieves a kappa coefficient of 80.34%, while traditional models such as U-Net (52.03%) and Attention-UNet (57.66%) show significantly lower consistency, further highlighting the advantages of Transformer models.

Lights-Transformer also excels in precision and recall, achieving 91.46% and 91.52%, respectively, which are the highest among all models. High precision indicates Lights-Transformer’s significant advantage in reducing false positives, while high recall demonstrates its strong capability in comprehensive landslide region identification. Although HRNet approaches Lights-Transformer in precision (90.82%), it shows a noticeable gap in recall (87.24%). Compared to traditional models such as U-Net and Attention-UNet, Lights-Transformer’s leading position in these metrics is even more prominent.

**Figure 5:**
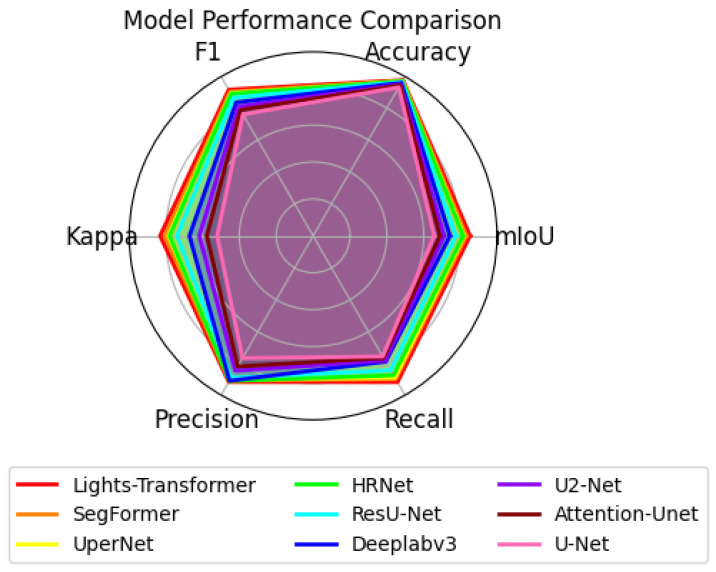
Radar chart comparing performance metrics of different models.

To provide a more intuitive comparison of the overall performance of different models in the landslide detection task, Figure 5 presents a comparison of Lights-Transformer with other mainstream models across several important evaluation metrics. The chart clearly shows that Lights-Transformer leads in all evaluation metrics, particularly in F1 score, accuracy, and mIoU, where it significantly outperforms other methods.

In conclusion, Lights-Transformer demonstrates outstanding all-around performance in landslide detection tasks.

**Figure 6:**
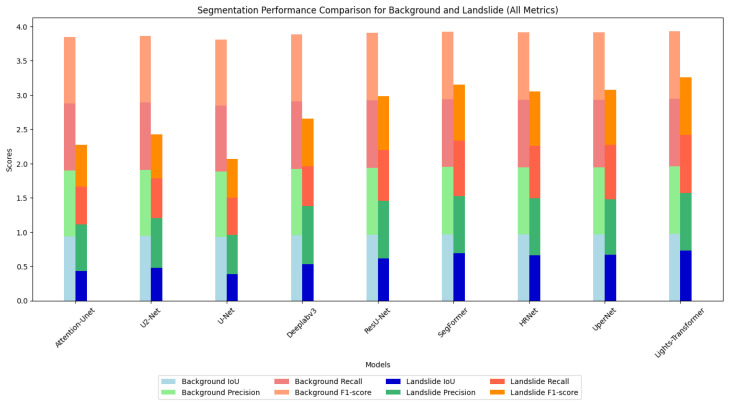
Segmentation performance comparison for background and landslide.

In the landslide detection task, analyzing the segmentation performance for landslide areas and background regions provides a more comprehensive evaluation of the model’s practical performance. Furthermore, Figure 6 presents a detailed comparison of the segmentation performance across various models for both background and landslide regions using bar charts. According to the results, the Lights-Transformer outperforms other models in both background and landslide regions, demonstrating a particularly significant advantage in segmentation accuracy for landslide areas. This figure vividly illustrates the exceptional capability of the Lights-Transformer in fine-grained segmentation tasks, especially in identifying complex boundaries and segmenting small targets.

Based on the IoU, precision, recall, and F1 score metrics in Table 6, the proposed Lights-Transformer model demonstrates significant advantages in both categories, particularly excelling in the segmentation of landslide areas. The segmentation quality of background regions is crucial for the overall task, since they occupy the majority of the image. Lights-Transformer achieves an IoU of 97.25% for background regions, which is the highest among all models. Its precision (98.62%) and recall (98.60%) are near perfect, and the F1 score reaches an impressive 98.61%. These results indicate that Lights-Transformer can accurately and comprehensively identify background regions. In comparison, other models perform similarly on background regions but fail to surpass Lights-Transformer. SegFormer achieves a background IoU of 96.88%, which is second only to Lights-Transformer with slightly lower precision (98.29%) and recall (98.54%). HRNet and UperNet record background IoUs of 96.61% and 96.59%, respectively, showing a notable performance gap compared to Lights-Transformer. ResU-Net achieved a background IoU of 96.20% and a precision of 97.85%, demonstrating superior performance among CNN architectures yet still falling short of the Lights-Transformer.

The segmentation performance of landslide regions is a key indicator of model effectiveness in landslide detection tasks. Compared to background regions, landslide segmentation is more challenging due to the complex boundaries and relatively small area. In this aspect, Lights-Transformer also excels, achieving an IoU of 72.96%, the highest among all models, significantly surpassing the second-best model, SegFormer (69.39%) with an improvement of 3.57 percentage points. This highlights its capability in capturing complex boundaries and segmenting small targets. The precision (84.30%) and recall (84.44%) of Lights-Transformer are also the highest, indicating that it not only predicts landslide regions accurately but also provides comprehensive coverage of the actual landslide areas. Its F1 score (84.37%) is substantially higher than other models, especially traditional ones like U-Net (55.86%) and Attention-Unet (60.78%) with performance improvements exceeding 20%. SegFormer achieves a landslide IoU of 69.39%, precision of 83.15%, recall of 80.74%, and F1 score of 81.93%, ranking just behind Lights-Transformer but slightly inferior in detail capture. HRNet and UperNet record landslide IoUs of 66.08% and 67.03%, respectively, with moderate precision and recall, but their F1 scores are about 4 percentage points lower than Lights-Transformer. ResU-Net achieved a landslide IoU of 61.50%, a precision of 84.50%, a recall of 73.50%, and an F1 score of 78.68%, demonstrating considerable competitiveness among CNN models but still slightly inferior to Transformer architectures in small-target and complex-boundary recognition.

In the segmentation of background regions, Lights-Transformer achieves the highest IoU (97.25%) and the best F1 score (98.61%), demonstrating exceptional reliability and accuracy. For landslide region segmentation, Lights-Transformer also leads with an IoU of 72.96% and an F1 score of 84.37%, showcasing outstanding small-target recognition and complex boundary-capturing capabilities. These results fully validate the comprehensive advantages of Lights-Transformer in landslide detection segmentation tasks, particularly in the detailed segmentation of landslide areas and accurate classification of global backgrounds. Compared to other Transformer-based models such as SegFormer, Lights-Transformer achieves further optimization in global modeling and boundary recognition capabilities. Its advantages over traditional convolutional network models are even more pronounced.

### 4.5. Confusion Matrix Analysis

To further evaluate the classification performance of different models in the landslide detection task, we computed and normalized the confusion matrices for each model. The confusion matrix provides an intuitive representation of how each model distinguishes between landslide and background classes, thereby revealing potential patterns of misclassification.

**Figure 7:**
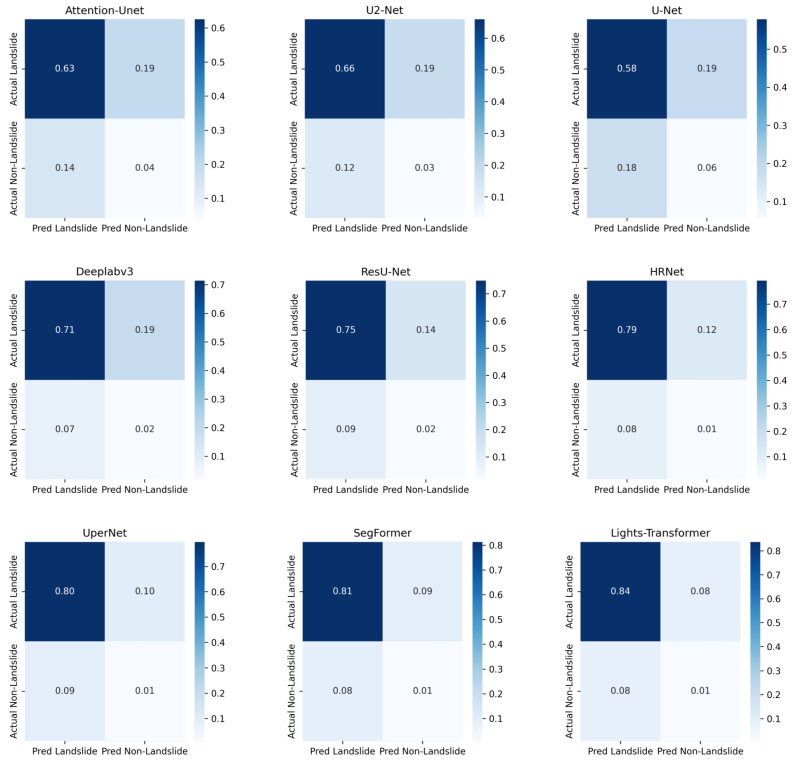
Confusion matrix analysis.

Figure 7 presents the normalized confusion matrices of each model on the test set. Each element in the normalized confusion matrix represents the probability that samples of a given class are predicted as a certain class, thereby mitigating the impact of class imbalance on the analysis.

Based on the analysis of the confusion matrices, Lights-Transformer achieved the best performance in recognizing landslide regions, demonstrating high TP rates and low FP and FN rates. This makes it well suited for applications requiring high landslide detection accuracy. Although DeepLabv3, ResU-Net and HRNet exhibit strong predictive capabilities for landslide areas, they suffer from relatively high false positives in non-landslide regions. In contrast, U-Net and U2-Net show weaker performance particularly with considerable false negatives and false positives in landslide regions. SegFormer and UperNet also demonstrate promising landslide detection capabilities, although they exhibit some degree of misclassification in non-landslide areas. Overall, model selection should be guided by the specific application requirements: lightweight models such as U-Net may be more appropriate in resource-constrained environments, while Lights-Transformer is preferable for scenarios demanding high precision.

### 4.6. Model Inference Analysis

To comprehensively evaluate the efficiency of different models in practical applications, we measured the average inference time (including forward propagation and result output) for processing a single 1024 × 1024 pixel image on the GDCLD test set. The comparison of inference times between Lights-Transformer and other mainstream models is summarized in Table 7.

Lights-Transformer demonstrates outstanding performance in terms of inference time, requiring only 103 ms, indicating high inference efficiency and making it suitable for applications with strict real-time requirements. Despite having a moderate model complexity of 183 G and 61 M parameters, it achieves a well-balanced trade-off between inference speed and computational cost.

In comparison, SegFormer exhibits a slightly longer inference time of 126 ms. However, with its higher model complexity (341 G) and 64 M parameters, it delivers enhanced capability in handling more complex tasks. Its architecture is evidently optimized for fine-grained and detailed feature extraction, which, although at the cost of speed, makes SegFormer advantageous in scenarios requiring high-precision feature analysis.

UperNet has the longest inference time among all models, reaching 193 ms, reflecting its substantial computational demand. With 93 M parameters and an extremely high model complexity of 2052 G, UperNet is suited for computationally intensive tasks and may outperform other models in terms of accuracy when deployed on high-performance computing platforms.

HRNet, with an inference time of 123 ms, strikes a balance between performance and computational load. It contains 70 M parameters and a complexity of 646 G. This balance makes HRNet a strong candidate for applications requiring relatively high precision without incurring excessive computational costs.

U-Net stands out with the shortest inference time of just 74 ms, making it particularly attractive for applications requiring rapid responses. Its low model complexity (497 G) and small parameter count (13 M) ensure minimal computational demand and memory usage, making it ideal for deployment in resource-constrained environments.

U2-Net, with an inference time of 97 ms, 44 M parameters, and a complexity of 603 G, offers a good compromise between inference efficiency and computational requirements. Although its inference time is slightly longer than U-Net, its relatively low complexity and parameter count make it well suited for tasks with limited computational resources but increased model demands.

Deeplabv3 achieves a relatively short inference time of 76 ms, making it suitable for fast-response applications. With a model complexity of 651 G and 39 M parameters, it exhibits a favorable balance between computational efficiency and memory consumption.

Attention-UNet, with an inference time of 120 ms, features a relatively low parameter count (35 M) and a model complexity of 1065 G. Although its inference time is longer, it shows efficient memory and computational usage, making it especially suitable for deployment in resource-constrained environments.

ResU-Net’s inference time was 115 milliseconds, with a model complexity of 385 G and a parameter count of 54 M, achieving a relatively balanced performance between inference efficiency and computational cost, making it a viable complementary choice as a lightweight model.

In summary, Lights-Transformer emerges as an ideal choice for scenarios requiring real-time performance with limited computational resources, owing to its fast inference time and moderate computational cost. In contrast, UperNet and SegFormer trade inference efficiency for greater capability in handling complex tasks, enabled by their higher model complexity and parameter count, making them well suited for deployment on high-performance computing platforms. For resource-constrained environments, U-Net and Attention-UNet offer a favorable compromise between inference speed and computational efficiency, delivering reasonable performance with low memory and computational overhead.

To comprehensively evaluate the practicality of each model in landslide detection tasks, we not only focused on segmentation accuracy metrics (such as mIoU and F1 score) but also compared the inference time of each model under the same hardware environment to further analyze their deployment potential in real-world scenarios. As shown in the table above, models such as UperNet and SegFormer demonstrated outstanding accuracy, but due to their complex structures, they exhibited inference times exceeding 120 ms, rendering them unsuitable for scenarios requiring high response speeds. In contrast, lightweight architectures such as U-Net and U2-Net achieved shorter inference times (less than 90 ms), but their segmentation accuracy was insufficient in complex terrain.

While ensuring leading accuracy (mIoU = 85.11%, F1 = 91.49%), Lights-Transformer maintains an inference time of 103 ms, achieving a favorable balance between speed and accuracy. This result indicates that Lights-Transformer possesses good “quasi-real-time” processing capabilities, offering support for practical applications such as post-disaster rapid response and emergency early warning in landslide scenarios.

### 4.7. Ablation Experiments

To validate the effectiveness of each key module in Lights-Transformer, we designed a systematic ablation experiment covering Efficient Self-Attention (ESA), Mix-FNN, Fusion Block, and Light Segmentation Head (LSH). The experimental results are shown in Table 8.

When all modules are fully enabled, the model achieves optimal performance (mIoU = 0.8511, precision = 0.9146, inference time = 103 ms), demonstrating a good balance between accuracy and efficiency. Sequentially removing each module reveals the following phenomena. After removing ESA, the mIoU drops to 0.8322, precision drops to 0.9027, and inference time significantly increases to 125 ms, indicating that the ESA module not only enhances global context modeling capability but also optimizes speed–structure coupling. After removing Mix-FNN, the mIoU drops to the lowest value of 0.8190 and precision declines to 0.8831, validating its critical role in spatial position awareness and structural modeling. After removing Fusion Block, the mIoU drops to 0.8237 and boundary performance weakens. Although the inference time slightly improves (99 ms), the overall semantic consistency deteriorates. After removing LSH, the mIoU drops to 0.8311 and inference time increases to 115 ms, indicating that this module achieves an effective trade-off between inference efficiency and semantic enhancement. In summary, each module provides strong support for the model’s performance with Mix-FNN and ESA playing pivotal roles in local and global information modeling, respectively. The final structure achieves a unified design of lightweight modules, leading accuracy, and deployable inference performance.

## 5. Analysis and Discussion

In the validation set, we randomly selected several remote sensing images for the visual analysis of landslide recognition. The figure below presents the input images (Input), ground truth labels, and the predictions of our model compared with other models. Black regions represent the background, while white regions indicate landslides.

**Figure 8:**
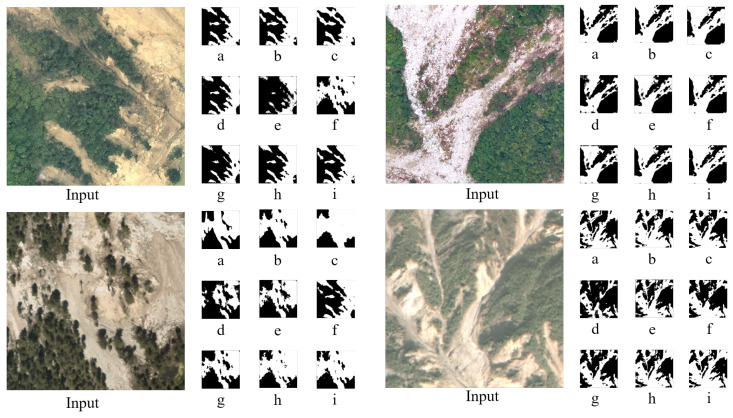
Prediction results comparison. (**a**) Ground truth labels; (**b**–**i**) the prediction results of Lights-Transformer, SegFormer, DeepLabv3, U-Net, U2-Net, Attention-Unet, and HRNet respectively.

From the analysis of Figure 8, it is evident that our model achieves a high degree of overlap with the ground truth, delineating landslide boundaries more clearly. Although in some cases it may not fully predict the landslide regions, the model consistently demonstrates high accuracy. This further validates the reliability of Lights-Transformer. Since the visualization results of ResU-Net are very similar to those of DeepLabv3, subsequent visualization figures will exclude ResU-Net.

To assess the generalization ability of the Lights-Transformer model across different geographic regions, we selected four representative landslide areas from the test set: Lushan, Palu, Mesetas, and Sumatra. The landslide prediction performance of the model was analyzed and compared across these regions. Table 9 presents the performance evaluation results of Lights-Transformer and other mainstream models in these distinct test areas. For each region, the landslide data are visualized in the figure, where red areas represent the ground truth landslide regions and yellow areas indicate the model’s predicted landslide regions.

Sumatra Region: Lights-Transformer achieved the highest values in mIoU, F1, and precision metrics, indicating its exceptional capability in landslide prediction under complex terrain conditions.

Palu and Mesetas Regions: Despite the complex topography and diverse landslide morphologies in these regions, Lights-Transformer maintained high consistency, demonstrating its adaptability to complex features.

Lushan Region: Lights-Transformer attained optimal performance in kappa and recall metrics, highlighting its outstanding ability to predict the completeness of landslide data in this region.

**Figure 9:**
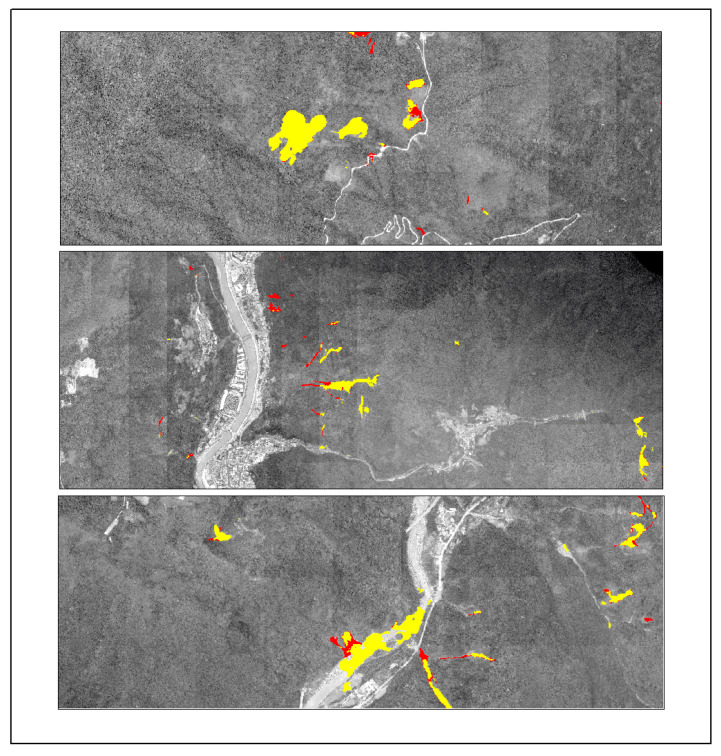
Lushan prediction result (Red: Ground-truth landslides; Yellow: Predicted landslides).

**Figure 10:**
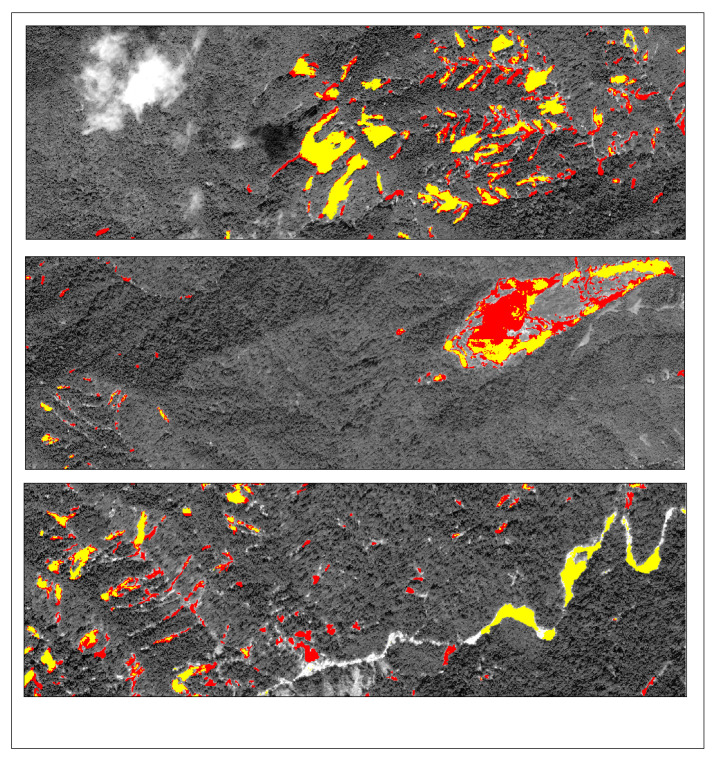
Palu prediction result (Red: Ground-truth landslides; Yellow: Predicted landslides).

**Figure 11:**
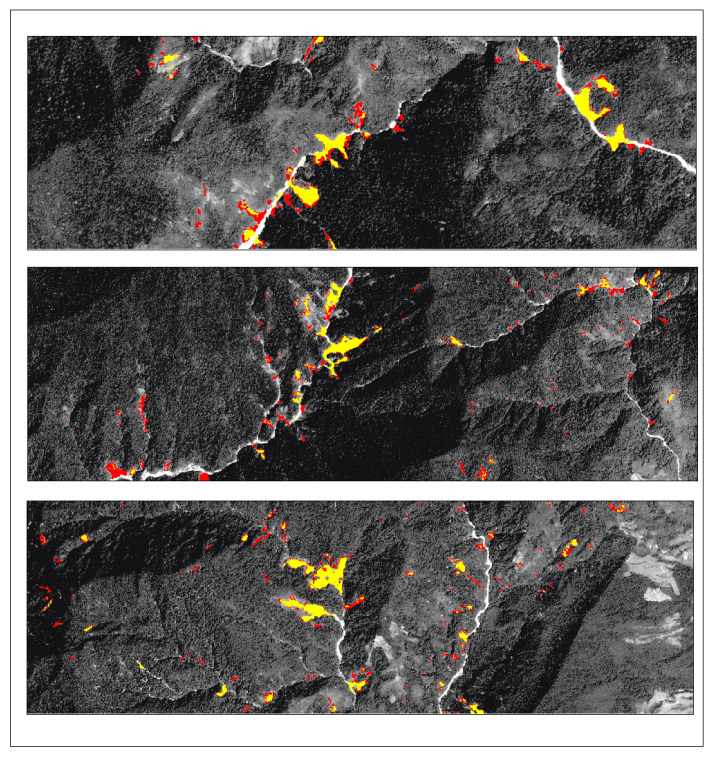
Mesetas prediction result (Red: Ground-truth landslides; Yellow: Predicted landslides).

**Figure 12:**
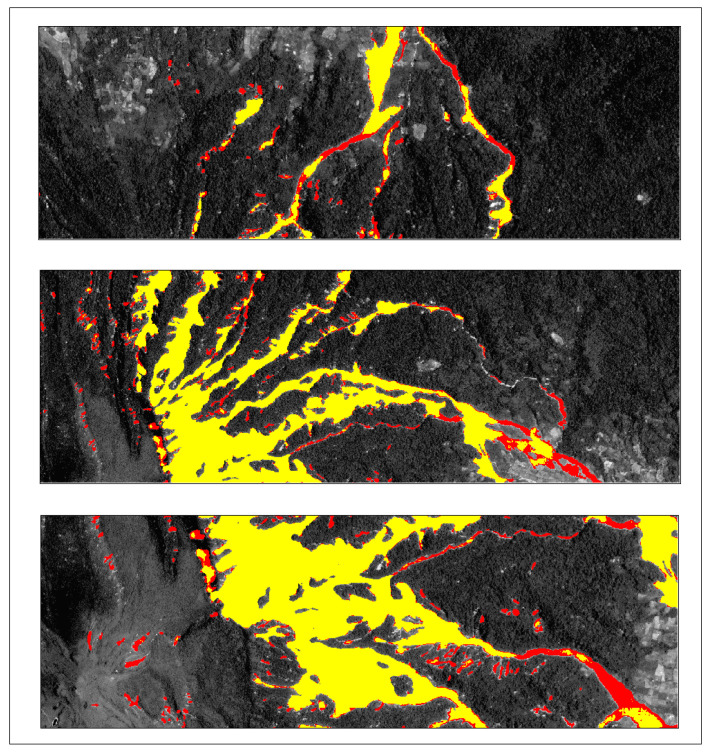
Sumarta prediction result (Red: Ground-truth landslides; Yellow: Predicted landslides).

Additionally, the visualized results of regional landslide predictions show that Lights-Transformer effectively aligns with actual landslide areas while significantly reducing false detections (as shown in Figure 9, Figure 10, Figure 11 and Figure 12).

The test results clearly indicate that Lights-Transformer achieves extremely high prediction accuracy and completeness for large, densely distributed landslide areas. In test regions such as Sumatra and Lushan, the overlap between the predicted areas and the true labels is very high. This demonstrates that Lights-Transformer has significant advantages in the feature extraction and global modeling of landslide regions. The Palu and Mesetas regions are characterized by complex topography and diverse landslide morphologies. In these regions, Lights-Transformer still maintains a high level of agreement between predicted landslide areas and true labels, showcasing its strong adaptability. The multi-head attention mechanism embedded in the Transformer architecture effectively captures detailed features of landslide areas at various scales, enhancing the model’s ability to adapt to diverse geomorphological characteristics. In complex terrains, there is often interference from large areas of non-landslide regions (such as vegetation, bare soil, etc.). Lights-Transformer, with its powerful feature representation capability, can reduce false detections to some extent. From the test results across multiple regions, Lights-Transformer shows high accuracy in predicting the boundaries of large-scale landslide areas.

Although the Lights-Transformer model demonstrates excellent performance across multiple evaluation metrics and achieves high-accuracy landslide detection in most test regions, it still faces some potential limitations in practical applications, particularly under varying terrain types and image resolution conditions. We observed that in complex background areas, such as hills and urban fringes, the model’s performance is slightly inferior to that in typical mountainous regions. For example, in the Palu and Mesetas regions, due to diverse land cover types and blurred landslide boundaries, Lights-Transformer achieved mIoU values of 0.6386 and 0.6597, respectively, which are both lower than its performance in regions with distinct geomorphological features such as Sumatra (mIoU = 0.7917). This indicates that the model still has certain limitations when handling areas with irregular terrain and mixed land cover. Although this study primarily used 1024 × 1024 image patches for training and testing, low-resolution images exhibited issues such as boundary blurring and small landslide target omissions during cross-regional transfer tests.

**Figure 13:**
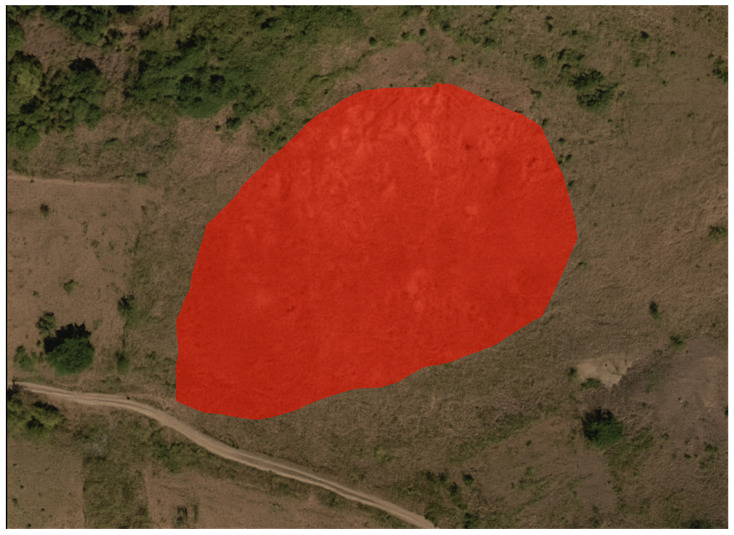
Bare soil misclassifed as landslide(regions marked in red correspond to false positives).

**Figure 14:**
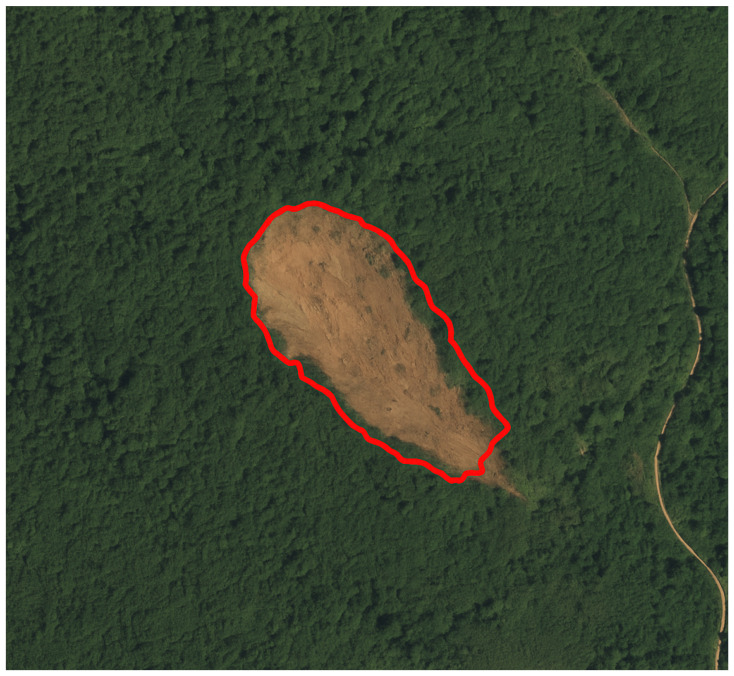
Omitted landslide area covered by vegetation(missed detections are marked with red bounding boxes).

As shown in Figure 13, the model exhibited typical false positives in non-landslide areas with similar texture or color features. In Figure 14, blurred image boundary information led to multiple small landslide regions not being accurately detected. These observations further illustrate that enhancing the model’s robustness remains a critical challenge particularly when dealing with complex backgrounds or low-resolution images.

## 6. Conclusions

This paper proposes a lightweight Transformer-based model for landslide detection, named Lights-Transformer, which incorporates a multi-scale feature fusion module (Fusion Block) and a lightweight segmentation head (Light Segmentation Head). The model achieves state-of-the-art segmentation performance on a publicly available earthquake-induced landslide dataset. Experimental results demonstrate that Lights-Transformer outperforms existing mainstream methods in terms of mIoU, F1 score, and inference efficiency, significantly reducing computational complexity while maintaining high accuracy. These features make it particularly suitable for landslide detection in high-resolution remote sensing imagery.

Despite its advantages, Lights-Transformer has certain limitations. For instance, the model relies heavily on high-resolution imagery, posing challenges such as increased memory consumption and limited adaptability to low-resolution data. Moreover, the model has not yet been systematically validated under extreme terrain and complex climatic conditions, and its generalization capability requires further enhancement. Future research will focus on the following directions: first, incorporating model pruning, knowledge distillation, and compression techniques to lower deployment costs; second, integrating terrain, temporal, and multi-source remote sensing data to improve adaptability in complex scenarios; and third, developing a lightweight deployment version of Lights-Transformer for broader applications in dynamic landslide monitoring and real-time early warning.

In summary, Lights-Transformer offers a novel and effective technical framework for landslide detection by balancing accuracy and computational efficiency. It also provides valuable insights for the design and optimization of intelligent segmentation models in the field of remote sensing.

## Figures and Tables

**Table 1:** Landslide events and their characteristics (all image patches are 1024 × 1024).

Events	Magnitudes	Time	Geographic Coordinates	Landslide Number	Total Landslide Area (km^2^)	Spatial Resolutions (m)
Jiuzhaigou	6.5	2017	(102.82° E, 33.20° N)	2498	14.5	0.5, 3
Mainling	6.4	2017	(95.02° E, 29.75° N)	1448	33.6	3
Hokkaido	6.6	2018	(142.01° E, 42.69° N)	7962	23.8	0.5, 3
Palu	7.5	2018	(119.84° E, 0.18° S)	15,700	43.0	1
Mesetas	6.0	2019	(76.19° W, 3.45° N)	804	8.5	3
Nippes	7.2	2021	(73.45° W, 18.35° N)	4893	45.6	0.5, 3
Sumatra	6.1	2022	(100.10° E, 0.22° N)	602	10.6	3
Lushan	5.9	2022	(102.94° E, 30.37° N)	1063	7.2	0.2, 0.5, 3
Luding	6.8	2022	(102.08° E, 29.59° N)	15,163	28.53	0.5, 2, 3

**Table 2:** Configuration parameters for the system.

Parameters	Configuration
CPU	Intel(R) Xeon(R) Gold 6226R
Operating System	Win11
GPU	NVIDIA GeForce RTX 3090
Deep Learning Architecture	PaddlePaddle

**Table 3:** Key parameters used during the experiment.

Parameters	Setup
batch_size	2
iters	30,000
optimizer	SGD
weight_decay	4.0 × 10^−5^
learning_rate	0.01
loss	CrossEntropyLoss

**Table 4:** Confusion matrix for landslide detection.

Ground Truth	Predicted Result	
	Landslide	Non-Landslide
**Landslide**	TP (True Positive)	FN (False Negative)
**Non-Landslide**	FP (False Positive)	TN (True Negative)

**Table 5:** Model performance metrics.

Model	mIoU	Accuracy	F1	Kappa	Precision	Recall
Attention-Unet	0.6877	0.9417	0.7881	0.5766	0.8176	0.7647
U2-Net	0.7108	0.9478	0.8086	0.6175	0.8445	0.7808
U-Net	0.6569	0.9295	0.7602	0.5203	0.7660	0.7548
Deeplabv3	0.7426	0.9576	0.8349	0.6707	0.9070	0.7875
ResU-Net	0.7750	0.9630	0.8650	0.7380	0.8890	0.8420
HRNet	0.8135	0.9682	0.8893	0.7786	0.9082	0.8724
UperNet	0.8181	0.9681	0.8926	0.7852	0.8971	0.8883
SegFormer	0.8313	0.9709	0.9017	0.8034	0.9072	0.8964
Lights-Transformer	**0.8511**	**0.9744**	**0.9149**	**0.8298**	**0.9146**	**0.9152**

**Table 6:** Comparison of segmentation accuracy for each category (landslide and background).

Model	Class	IoU	Precision	Recall	F1-Score
Attention-Unet	background	0.9389	0.9609	0.9762	0.9685
	landslide	0.4365	0.6744	0.5531	0.6078
U2-Net	background	0.9452	0.9634	0.9804	0.9718
	landslide	0.4763	0.7255	0.5811	0.6453
U-Net	background	0.9263	0.9598	0.9637	0.9617
	landslide	0.3875	0.5721	0.5458	0.5586
Deeplabv3	background	0.9555	0.9640	**0.9908**	0.9772
	landslide	0.5298	**0.8501**	0.5843	0.6926
ResU-Net	background	0.9620	0.9785	0.9850	0.9817
	landslide	0.6150	0.8450	0.7350	0.7868
SegFormer	background	0.9688	0.9829	0.9854	0.9842
	landslide	0.6939	0.8315	0.8074	0.8193
HRNet	background	0.9661	0.9786	0.9870	0.9828
	landslide	0.6608	0.8378	0.7578	0.7958
UperNet	background	0.9659	0.9816	0.9837	0.9827
	landslide	0.6703	0.8125	0.7929	0.8026
Lights-Transformer	background	**0.9725**	**0.9862**	0.9860	**0.9861**
	landslide	**0.7296**	0.8430	**0.8444**	**0.8437**

**Table 7:** Comparison of models based on inference time, computational complexity, and parameters.

Model	Average Inference Time (ms)	Model Complexity (G)	Number of Parameters (M)
Lights-Transformer	103	**183**	61
SegFormer	126	341	64
UperNet	193	2052	93
HRNet	123	646	70
U-Net	**74**	497	**13**
U2-Net	97	603	44
Deeplabv3	76	651	39
Attention-Unet	120	1065	35
ResU-Net	115	385	54

**Table 8:** Ablation study of key components in Lights-Transformer.

ESA	Mix-FNN	Fusion Block	LSH	mIoU	Precision	Inference Time (ms)
✗	✓	✓	✓	0.8322	0.9027	125
✓	✗	✓	✓	0.8190	0.8831	101
✓	✓	✗	✓	0.8237	0.8962	**99**
✓	✓	✓	✗	0.8311	0.9035	115
✓	✓	✓	✓	**0.8511**	**0.9146**	103

**Table 9:** Model metrics for Lushan, Palu, Mesetas, and Sumatra.

Region	Model	mIoU	Accuracy	F1	Kappa	Precision	Recall
Lushan	Lights-Transformer	**0.7405**	**0.9976**	**0.8253**	**0.6505**	0.8651	**0.7933**
	SegFormer	0.6754	0.9974	0.7604	0.5211	**0.9549**	0.6828
	UperNet	0.6867	0.9974	0.7727	0.5455	0.9141	0.7036
	HRNet	0.6943	0.9970	0.7806	0.5612	0.8203	0.7496
Palu	Lights-Transformer	**0.6386**	0.9893	**0.7209**	**0.4422**	0.8007	**0.6752**
	SegFormer	0.5984	0.9890	0.6693	0.3398	0.8164	0.6168
	UperNet	0.6093	**0.9900**	0.6835	0.3686	**0.9025**	0.6202
Mesetas	Lights-Transformer	**0.6597**	**0.9954**	**0.7436**	**0.4876**	**0.9186**	**0.6724**
	SegFormer	0.5614	0.9941	0.6125	0.2263	0.8532	0.5676
Sumatra	Lights-Transformer	**0.7917**	**0.9953**	**0.8691**	**0.7383**	0.9246	**0.8266**
	SegFormer	0.7748	0.9952	0.8555	0.7111	**0.9584**	0.7908

## Data Availability

The GDCLD landslide dataset is freely available at https://doi.org/10.5281/zenodo.13612636 accessed on 24 October 2024.
